# Local Ancestry Inference Based on Population-Specific Single-Nucleotide Polymorphisms—A Study of Admixed Populations in the 1000 Genomes Project

**DOI:** 10.3390/genes15081099

**Published:** 2024-08-21

**Authors:** Haoyue Fu, Gang Shi

**Affiliations:** School of Telecommunications Engineering, Xidian University, 2 South Taibai Road, Xi’an 710071, China; haoyuefu@stu.xidian.edu.cn

**Keywords:** human populations, admixture, local ancestry inference, population-specific SNPs

## Abstract

Human populations have interacted throughout history, and a considerable portion of modern human populations show evidence of admixture. Local ancestry inference (LAI) is focused on detecting the genetic ancestry of chromosomal segments in admixed individuals and has wide applications. In this work, we proposed a new LAI method based on population-specific single-nucleotide polymorphisms (SNPs) and applied it in the analysis of admixed populations in the 1000 Genomes Project (1KGP). Based on population-specific SNPs in a sliding window, we computed local ancestry information vectors, which are moment estimators of local ancestral proportions, for two haplotypes of an admixed individual and inferred the local ancestral origins. Then we used African (AFR), East Asian (EAS), European (EUR) and South Asian (SAS) populations from the 1KGP and indigenous American (AMR) populations from the Human Genome Diversity Project (HGDP) as reference populations and conducted the proposed LAI analysis on African American populations and American populations in the 1KGP. The results were compared with those obtained by RFMix, G-Nomix and FLARE. We demonstrated that the existence of alleles in a chromosomal region that are specific to a particular reference population and the absence of alleles specific to the other reference populations provide reasonable evidence for determining the ancestral origin of the region. Contemporary AFR, AMR and EUR populations approximate ancestral populations of the admixed populations well, and the results from RFMix, G-Nomix and FLARE largely agree with those from the Ancestral Spectrum Analyzer (ASA), in which the proposed method was implemented. When admixtures are ancient and contemporary reference populations do not satisfactorily approximate ancestral populations, the performances of RFMix, G-Nomix and FLARE deteriorate with increased error rates and fragmented chromosomal segments. In contrast, our method provides fair results.

## 1. Introduction

Population admixture has occurred throughout the long history of human evolution [1]. This process results in the transfer of genetic materials and the creation of admixed populations [2]. In admixed populations, genomes are composed of chromosomal segments from different ancestral populations [3]. Local ancestry inference (LAI) uses reference populations and a range of statistical models to infer the ancestral origins of chromosomal segments in admixed individuals [4]. It is an important tool in population evolution and historical studies [5]. In genome-wide association studies (GWASs), LAI is used to partition the genomes of admixed samples into distinct ancestral chromosomal segments, allowing them to be integrated into GWASs [6]. LAI is also employed in admixture mapping to examine the association between local ancestry and disease traits in admixed populations [7]. However, the process of population admixture is complex and often protracted [1,8,9], and it is challenging to conduct LAI for admixed individuals.

A number of LAI methods have been developed. Early methods, such as STRUCTURE, were based on a Bayesian inference framework that inferred local ancestry using the Hidden Markov Model (HMM) [10]. Later, ADMIXMAP and ANCESTRYMAP, which adopted similar Bayesian inference frameworks, were proposed [11,12]. LAMP uses a clustering algorithm to estimate the ancestral origin in a sliding window [13]. SupportMix, a machine learning-based method, was proposed to infer locus-specific genomic ancestry when many possible ancestral populations are analyzed simultaneously [14]. RFMix uses a conditional random field framework parameterized by random forests to estimate ancestry with improved performance by learning from admixed samples [15]. It is currently the most widely used method and is currently considered the “gold standard” [4,16]. ELAI and LOTER, methods based on extensions of HMM, were subsequently developed [17,18]. ELAI uses diploid data, so phase uncertainty is eliminated [17]. LOTER, taking an optimization perspective, uses dynamic programming to obtain optimal solutions [18]. Brown and Pasaniuc proposed Lanc-CSV, which combined continental population-specific variants with the HMM [19], demonstrating higher accuracy and efficiency. More recently, G-Nomix used supervised learning to train models using haplotype data from reference populations [20]. FLARE achieved high inference precision using an extended Li and Stephens model [21]. Notably, in the 1000 Genome Project (1KGP) dataset released in phase 3, approximately 86% of the 88 million polymorphisms are specific to a single continental population [22]. Population-specific SNPs could play an important role in characterizing population ancestry and investigating population history [23], however, there lack analytical methods and tools to explore them effectively.

Based on population-specific SNPs, we recently proposed the ancestral information vector, the best linear unbiased estimator of ancestral proportions, to conduct global ancestry inference for admixed individuals [24]. In the current work, we extend the probabilistic framework in [24] to a local ancestry information vector (LAIV), which forms the basis for the LAI method proposed in this paper. The new method was implemented in program “lai” and included in the software package Ancestral Spectrum Analyzer (ASA) version 1.2.0, which is accessible at https://github.com/eat1000/ASA, accessed on 15 June 2024. Then we applied the method to analyze African American (AA) populations (*N* = 157) and American populations (*N* = 347) in the 1KGP [25] and compared the results with those obtained with RFMix, G-Nomix and FLARE. We used five continental populations as reference populations: African (AFR), East Asian (EAS), European (EUR) and South Asian (SAS) populations from the 1KGP [25] and indigenous American (AMR) populations from the Human Genome Diversity Project (HGDP) Centre d’Etude du Polymorphisme Humain (CEPH) panel [26]. We show that the results of RFMix, G-Nomix and FLARE are highly consistent with those of ASA, which are also consistent with the distributions of population-specific alleles carried by admixed individuals. Contemporary populations serve as proxies for ancestral populations well when recent admixtures are inferred. In the analysis of individuals close to AMR populations, in which case contemporary reference populations do not satisfactorily approximate ancestral populations, ASA produced more reasonable results than the other three methods in the inference of ancient admixtures when using AFR, EAS, EUR and SAS reference populations.

## 2. Materials and Methods

### 2.1. Local Ancestry Information Vector

Consider *N* admixed individuals whose haplotype segment contains *M* diallelic population-specific SNPs from *K* reference populations. Let $\theta_{k}$ be the index set of SNPs specific to reference population *k* and $M_{k}$ the number of SNPs specific to population *k*. Then, the total number of SNPs is $M=M_{1}+M_{2}+\ldots +M_{K}$. Let $f_{km}$ represent the minor allele frequency (MAF) of SNP *m* in reference population *k*. It is evident that $f_{km}$ must be greater than zero if *m* is in the index set $\theta_{k}$ and zero otherwise.

A haplotype matrix *X* of dimension N×M can be defined, whose element $X\left(n,\;m\right)$ indicates whether the population-specific allele of SNP *m* is present in the haplotype segment for individual *n*. Were individual *n* from reference population *k*, $X\left(n,\;m\right)$ would follow a binomial distribution that takes values of 0 and 1 with probabilities $1-f_{km}$ and $f_{km}$, respectively, provided that $m\;\in \;\theta_{k}$. Consider individual *n* whose ancestral populations are in the *K* reference populations. Let $p_{n}^{1},p_{n}^{2},\;\ldots ,\;p_{n}^{K}$ denote the ancestral proportions of individual *n* in the haplotype segment. That is, $p_{n}^{k}$ is the probability with which the haplotype segment comes from population *k*.

Assuming the independence of the *M* SNPs in the reference populations, $X\left(n,\;1\right)\;,\;\ldots ,\;X\left(n,\;M\right)$ have the following distribution:

(1) $$p\left(X\left(n,\;1\right)\;,\;\ldots ,\;X\left(n,\;M\right)\right)\;=\prod_{k=1}^{K}\prod_{m\;\in \;\theta_{k}}{\left(p_{n}^{k}f_{km}\right)}^{X\left(n,m\right)}{\left(1-p_{n}^{k}f_{km}\right)}^{1-X\left(n,m\right)},$$

where $X\left(n,m\right)=0,\;1$, *m* = 1, 2, …, *M*. Following similar arguments in [24], it can be shown that the estimate of $p_{n}^{k}$ by the method of moment is

(2) $${\hat{p}}_{n}^{k}=\frac{\sum_{m\;\in \;\theta_{k}}X\left(n,\;m\right)}{\sum_{m\;\in \;\theta_{k}}f_{km}},$$

where *k* = 1, 2, …, *K*. The numerator is the observed number of minor alleles specific to population *k*, and the denominator is the sum of the MAFs of the SNPs. In fact, the estimator (2) does not depend on the assumption of independence among SNPs and works for the case with linkage disequilibrium as well. We define the LAIV for individual *n* in the haplotype segment as $C_{n}=\left[{\hat{p}}_{n}^{1},\;{\hat{p}}_{n}^{2},\ldots {\hat{p}}_{n}^{K}\right]$.

### 2.2. Local Ancestry Inference

For each of the population-specific SNPs, we considered a window of *d* base pairs (bps) centered at the SNP locus. Based on the haplotype data of individual *n* in the window, we computed two LAIVs for the two haploids. For each haploid, ancestry at the locus is determined as the population with the most ancestral information ${\hat{p}}_{n}^{k}$. As the window moves from one end of a chromosome to the other end, the ancestries of two haploids at the loci of population-specific SNPs are obtained. For loci between two SNPs whose ancestries coincide, ancestry is deemed to be the same. Otherwise, the ancestries of the loci are uncalled. To allow for ancestry calling with different levels of confidence, the algorithm implemented in ASA provides the option “--laiv-min”, which specifies the minimum value of the largest ancestral information for calling an ancestry. For a locus whose largest ancestral information is smaller than the threshold, its ancestry will not be called.

### 2.3. Reference Populations and Admixed Populations from the 1KGP and HGDP

We used genotype data from the 1KGP provided by the New York Genome Center (NYGC) [25]. The 2504 individuals from the 1KGP represent five continental populations: African (AFR), American, East Asian (EAS), European (EUR) and South Asian (SAS). The HGDP-CEPH panel, comprising 929 individuals, encompasses genotype data from 54 populations worldwide [26]. The 929 individuals represent seven geographic regions: Africa, America, Central and South Asia, East Asia, Europe, the Middle East and Oceania. Four continental populations from the 1KGP were selected as reference populations, namely, AFR (*N* = 504), EAS (*N* = 504), EUR (*N* = 503) and SAS (*N* = 489). In addition, we used indigenous American (AMR) populations (*N* = 61) from the HGDP-CEPH panel as the reference population for AMR. After removing SNPs with multicharacter alleles and inconsistent alleles, we merged the genotype dataset of AMR populations from the HGDP with the genotype dataset from the 1KGP using PLINK version 1.9 [27]. The combined dataset includes 109,574,699 diallelic SNPs, 2061 samples from the five reference populations (AFR, AMR, EAS, EUR, SAS) and 504 samples from the admixed populations, AAs and Americans in the 1KGP.

### 2.4. Population-Specific SNPs

Population-specific SNPs were selected using the Population-Specific SNP Screener (PSNPS) in ASA version 1.2.0. We considered SNPs with no missing genotype data in the reference populations. For each of the reference populations, we chose SNPs that had at least five copies of minor alleles and no such alleles in the other reference populations. This resulted in 5,793,448, 55,413, 928,153, 438,747 and 743,954 SNPs that are specific to the AFR, AMR, EAS, EUR and SAS populations, respectively. The panel of population-specific SNPs is enclosed in the Supplementary Files (https://doi.org/10.6084/m9.figshare.26027158).

### 2.5. Statistical Phasing

We selected SNPs with no missing genotype data and MAFs larger than 0.001 in the merged dataset using PLINK version 1.9 [27], which yielded 18,667,892 autosomal SNPs. We used SHAPEIT version 4.2.2 [28] to phase the filtered genotype data by chromosome with default parameters and genetic maps provided by the software. For the subsequent analysis, we split the haplotype data into two datasets using VCFtools version 0.1.17 [29]: one included samples from the reference populations, and the other included samples from the admixed populations.

### 2.6. Local Ancestry Analyses

We illustrate the analysis flow followed by this work in a diagram shown in Supplementary Figure S1. The parameters used in the analyses with ASA, RFMix, G-Nomix and FLARE are described as follows. The analyses with ASA version 1.2.0 were conducted with the options “--window-size” of 2 million bps, “--laiv-min” of 0.000001, and “--allele-min” of 2. The remaining parameters were defaults. The analyses with RFMix [15] version 2.03-r0 were conducted on the haplotype datasets of the admixed populations and the reference populations. The following parameters were used: “-n” was 5 for the five ancestral populations, the number of EM iterations “-e” was 1, and “--reanalyze-reference” was used to reanalyze and reclassify the local ancestry of the reference panel. The analyses with G-Nomix [20] were performed on the admixed populations with models trained with haplotype data of the reference populations. The default values of the options were used, and “phase” was specified as false for not correcting possible phasing errors using the ancestry predicted by G-Nomix. This combination of parameters will yield LAI results based on the same haplotype data used by the other methods. The analyses with FLARE version 0.3.0 [21] were carried out with the options “min-mac” and “min-maf” to be zeros in order to include all SNPs in the analyses, and “em” was true.

## 3. Results

In the 1KGP dataset, AA populations, which include African Caribbean in Barbados (ACB) and African Ancestry in Southwest US (ASW), and American populations, which include Colombian in Medellin (CLM), Mexican Ancestry in Los Angeles (MXL), Peruvian in Lima (PEL) and Puerto Rican in Puerto Rico (PUR), are known admixed populations [22]. Their demographic histories and population admixtures have been well studied [30,31,32,33]. AAs are of primarily African and European ancestries [30,32,33], and Americans are of European, African and indigenous American ancestries [31,32,33]. We analyzed AAs and Americans in the 1KGP with ASA, RFMix, G-Nomix and FLARE using AFR, AMR, EAS, EUR and SAS as the reference populations. Local ancestry results for the 504 individuals are provided in the Supplementary Files (https://doi.org/10.6084/m9.figshare.26027158), and consistency rates among the four methods are presented in Table 1.

### 3.1. African Americans in the 1KGP

**Figure 1:**
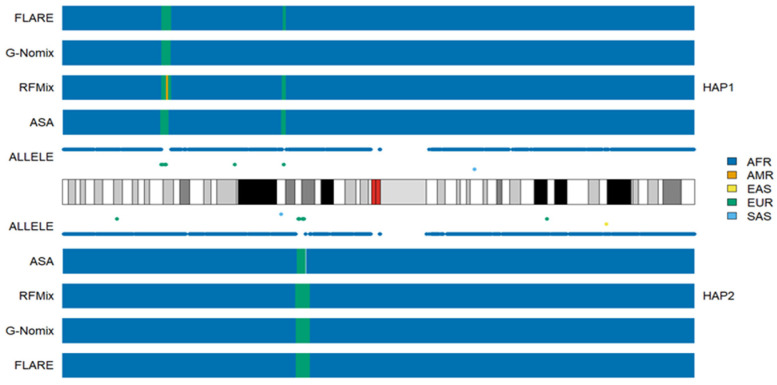
Local ancestry results of HG02052 on chromosome 1 with reference populations from AFR, AMR, EAS, EUR and SAS.

The local ancestry of an individual, HG02052 on chromosome 1, together with the population-specific alleles carried by this individual, are presented in Figure 1. HG02052 is from the ACB population, who is a typical African American; her global ancestral spectrum based on genotype data on 22 autosomes comprises AFR and EUR, and the ancestral information is 0.991 and 0.033, respectively [24]. The results of the four methods are approximately identical. In the broad chromosomal regions with dense alleles specific to AFRs, all four methods identified them as being of AFR ancestry. In the 1p34.3-1p34.2 region of haplotype 1, there is a chromosomal segment in which HG02052 carries alleles specific to EUR only, and a local ancestry of EUR was correspondingly called. Similarly, a segment in the 1p22.1-1p21.3 region of haplotype 2 was identified as EUR. The segment length called by ASA is shorter than that called by the other three methods. This is probably due to the influence of the first AFR allele on the right of the region, which is close to the cluster of EUR alleles and isolated from the cluster of AFR alleles further away. In the 1p22.3 region of haplotype 1, there are two alleles specific to EUR and no other population-specific alleles. The local ancestry of EUR was called by three methods except for G-Nomix. In centromeric regions, where SNP data are not available, local ancestries were probably inferred based on the results in adjacent regions. Notably, one EAS allele, two EUR alleles and two SAS alleles were distributed sporadically on chromosome 1. The presence of nearby AFR alleles did not affect ancestral calling in these regions. These five alleles probably came from misclassification errors in the process of screening population-specific SNPs, due to the limited sample sizes of the reference populations [24].

**Figure 2:**
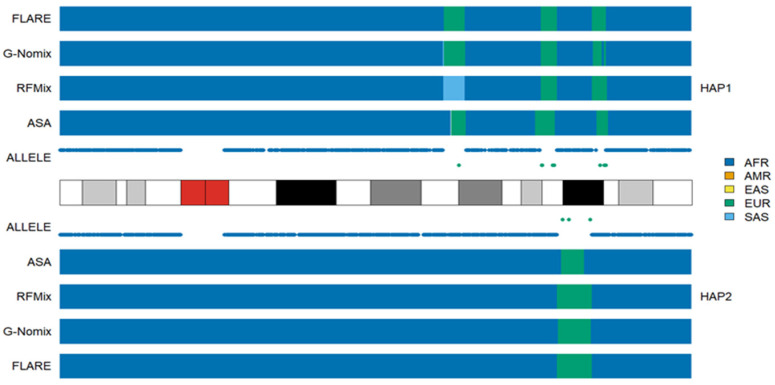
Local ancestry results of HG02330 on chromosome 18 with reference populations from AFR, AMR, EAS, EUR and SAS.

In the AA populations, the local ancestry results of RFMix, G-Nomix and FLARE were largely consistent with the distributions of population-specific alleles and thus with the results of ASA. The rates of consistency with the ASA were 0.963, 0.973, and 0.969 in the ACB population, respectively, and 0.941, 0.951, and 0.948 in the ASW population, respectively. The full consistency rates among the four methods are presented in Supplementary Figures S2 and S3 for ACB and ASW populations, respectively. Inconsistency occurs occasionally, and an example from another typical African American HG02330 is shown in Figure 2. In the 18q21.1-18q21.2 region of haplotype 1, RFMix called a segment as being of SAS ancestry, while G-Nomix and FLARE suggested EUR ancestry. There are five alleles specific to EUR carried by HG02330 and no alleles specific to SAS in the region. In accordance with the population-specific alleles, ASA detected the region as being of EUR ancestry.

**Figure 3:**
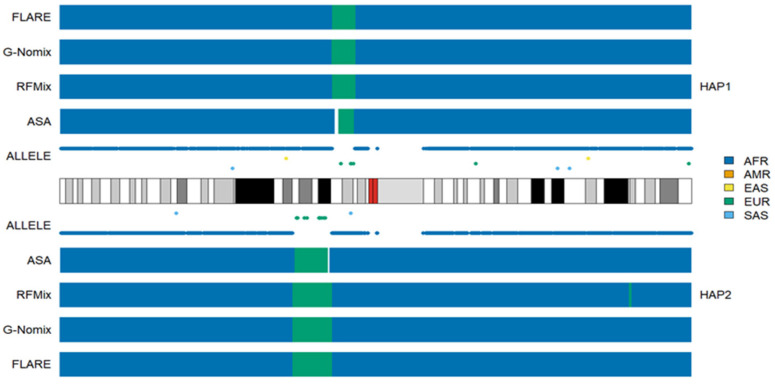
Local ancestry results of HG02107 on chromosome 1 with reference populations from AFR, AMR, EAS, EUR and SAS.

Because ASA determines local ancestries solely based on the distributions of population-specific alleles, its performance deteriorates in the boundaries of chromosomal segments that originate from different ancestries if such alleles are lacking in the region. Figure 3 illustrates the issue with the results for an individual HG02107, on chromosome 1. In the 1p13.3-1p13.1 region of haplotype 1, there are only six EUR alleles in the region of approximately 9 M bps, and the gap between the last AFR allele on the left and the first EUR allele is more than 3 M bps. ASA was not able to determine the ancestry for some of the gap regions, and there is an uncalled segment (white). The EUR segment called by ASA is also shorter than those called by RFMix, G-Nomix or FLARE. Similar phenomena can be observed for the EUR segment in the 1p22.1-1p13.3 region of haplotype 2. In contrast, RFMix, G-Nomix and FLARE make use of haplotype data estimated by all SNPs in the regions and provide local ancestry inferences. Average uncalled rates by ASA in ACB and ASW populations were 0.009 and 0.017, respectively.

**Figure 4:**
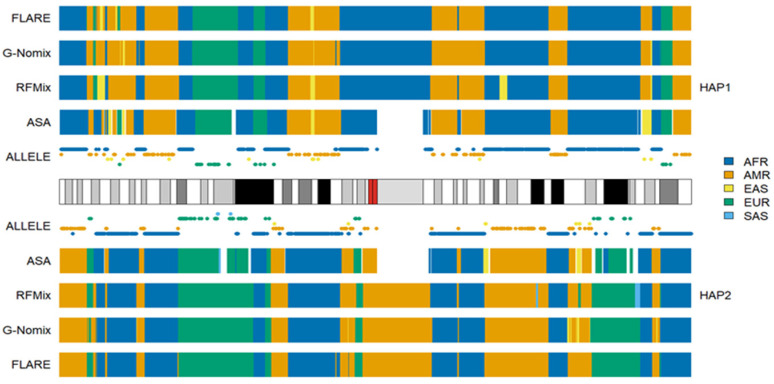
Local ancestry results of NA20299 on chromosome 1 with reference populations from AFR, AMR, EAS, EUR and SAS.

Compared with the ACB population, which is primarily of AFR and EUR ancestries, the ASW population has additional AMR ancestry. The results of an individual, NA20299, from ASW are shown in Figure 4. The admixture history of AAs is relatively recent, can be traced to a few hundred years ago and occurred in tens of generations [32,33]. The sums of ancestral information from AFR, AMR and EUR were close to the expected 1 in AA populations, which indicated that contemporary AFR, AMR and EUR populations approximated the ancestral populations of AAs well [24]. The local ancestry analysis results of the four methods were similar and consistent with the distributions of population-specific alleles carried by admixed individuals. This further suggests that contemporary AFR, AMR and EUR populations perform reasonably well as proxies for the ancestral populations of AAs.

### 3.2. Americans in the 1KGP

**Figure 5:**
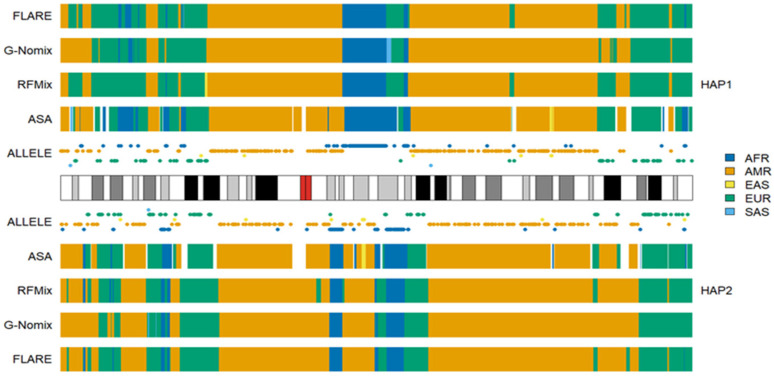
Local ancestry results of HG01893 on chromosome 2 with reference populations from AFR, AMR, EAS, EUR and SAS.

The ancestral components of Americans in the 1KGP are largely AFR, EUR and AMR, and their admixture histories are relatively recent as well. Ancestral proportions vary significantly across the CLM, MXL, PEL, and PUR populations and across individuals within the populations [24,31]. As in the analyses of AAs, the AFR, EUR and AMR populations in the reference panel approximate ancestral populations of Americans well. Figure 5 shows the local ancestry results of an individual HG01893, from the PEL population, on chromosome 2. Because the number of SNPs specific to AMR was only 55,413 on 22 autosomes, which is much smaller than the number of SNPs specific to the other four populations, AMR alleles were sparsely distributed in the chromosomal segments originating from AMR. As a result, ASA was not able to call some of the AMR regions due to the lack of AMR alleles. The average uncalled rates in CLM, MXL, PEL, and PUR were 0.073, 0.072, 0.044, and 0.073, respectively, which are greater than those in AAs. The consistency rates between ASA and the other three methods decreased slightly; details can be found in Supplementary Figures S4–S7 for CLM, MXL, PEL and PUR populations, respectively.

Interestingly, individual HG02272 from PEL has almost exclusive ancestry from AMR, with global AMR ancestral information of 0.931 [24]. HG02272 is closer to the indigenous AMRs in the HGDP than to the admixed Americans in the 1KGP. In the ancestral spectrum analysis of the indigenous AMRs in the HGDP with four reference populations, AFR, EAS, EUR and SAS from the 1KGP populations, it was shown that HGDP AMRs have average ancestral information of 0.289 and 0.065 from EAS and EUR, respectively [24]. This suggested that the ancestors of indigenous AMRs may have experienced admixture events in their demographic history. The admixtures might have dated back to sometime before the migration of AMR ancestors from Eurasia to America [24]. This was further supported by the ancestral spectrum analysis of ancient DNA AHUR_2064, which was obtained from Nevada, USA, approximately 10,970 years before present. AHUR_2064 showed exclusive coancestry with indigenous AMRs from the HGDP when analyzed with five reference populations, AFR, AMR, EAS, EUR and SAS. According to the analysis of four reference populations, AFR, EAS, EUR and SAS from the 1KGP, AHUR_2064 shares ancestry with EAS and EUR, with ancestral information of 0.189 and 0.024, respectively [34]. Hence, the admixture must have been ancient, occurring at least hundreds of generations ago. Obviously, contemporary EAS and EUR populations are not satisfactory proxies for ancestral populations of contemporary AMRs or AHUR_2064. Therefore, it is crucial to include an AMR reference population for the LAI of HG02272. However, analyzing HG02272 with four reference populations, AFR, EAS, EUR and SAS, offers a valuable opportunity to evaluate the performances of different methods in real data analysis of ancient admixture when reference populations do not satisfactorily approximate ancestral populations.

**Figure 6:**
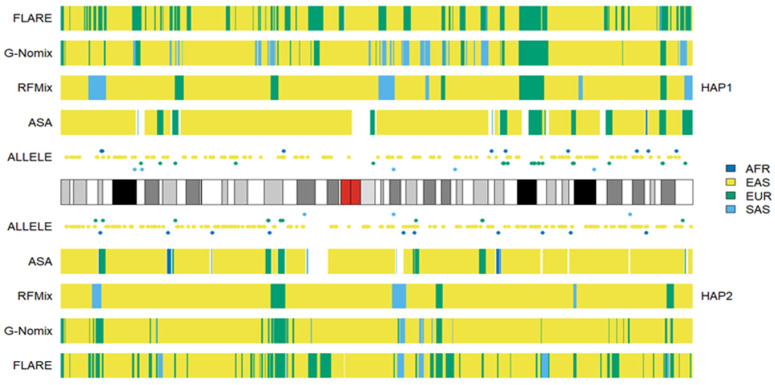
Local ancestry results of HG02272 on chromosome 3 with reference populations from AFR, EAS, EUR and SAS.

**Figure 7:**
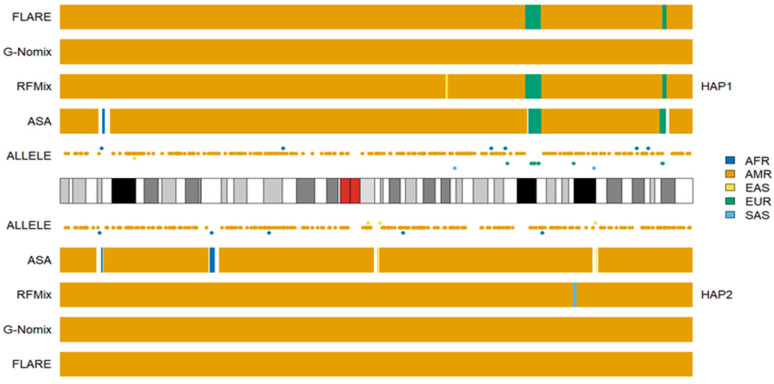
Local ancestry results of HG02272 on chromosome 3 with reference populations from AFR, AMR, EAS, EUR and SAS.

We conducted additional LAI with the four reference populations from the 1KGP, and the results for HG02272 on chromosome 3 are shown in Figure 6. Some SAS ancestries were called by RFMix, G-Nomix and FLARE but not by ASA. Considering that HG02272 carried only 7 alleles specific to SAS on chromosome 3, the SAS ancestries identified by RFMix, G-Nomix and FLARE are unlikely to be accurate. Some SAS populations were admixed with EAS and EUR [24]. RFMix, G-Nomix and FLARE might mistakenly classify some AMR haplotypes, which should originate from EAS or EUR, as being SAS due to admixtures in SAS reference populations. In contrast, the admixtures in the SAS reference populations will cause some SNPs that are specific to EAS or EUR to be undetected because they are also polymorphic in SAS reference populations. Hence, this will not result in classifying SNPs that are specific to EAS or EUR as being specific to SAS erroneously. In addition, G-Nomix and FLARE tended to yield many more chromosomal fragments of smaller sizes than ASA and RFMix. These ancestry calls are not sufficiently supported by the distributions of population-specific alleles. The consistency rates between the analysis of HG02272 by ASA and the other three methods, RFMix, G-Nomix and FLARE, were 0.802, 0.773 and 0.709, respectively. These results are consistent with those of simulation studies, which suggest that the performance of RFMix deteriorates when the admixtures become more ancient [18]. The results obtained using the five reference populations that included AMR are shown in Figure 7. Because HG02272 is close to the AMR population, all four methods performed well, and the differences among the results were minor. The consistency rates between ASA and RFMix, G-Nomix, and FLARE were 0.971, 0.979 and 0.981, respectively. AMR individuals from the HGDP were analyzed using the four reference populations from the 1KGP, and similar patterns were confirmed.

### 3.3. The Outliers in the 1KGP

**Figure 8:**
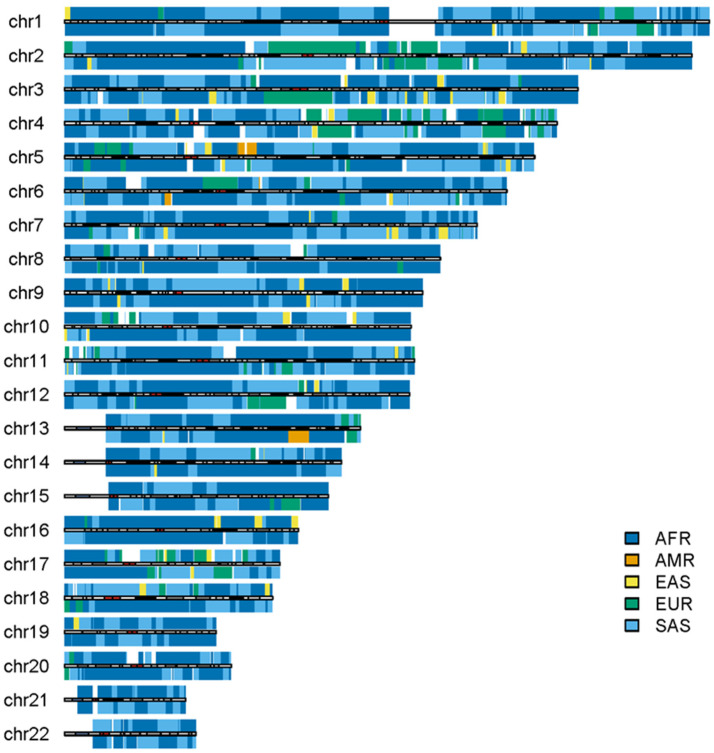
Local ancestry results of HG01880 on autosomes with reference populations from AFR, AMR, EAS, EUR and SAS. The analysis was conducted by ASA.

**Figure 9:**
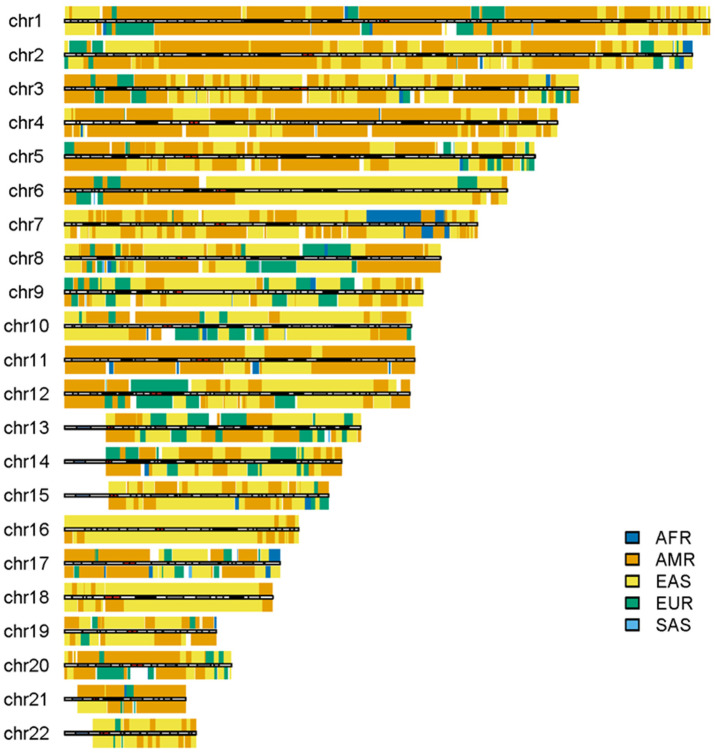
Local ancestry results of HG01944 on autosomes with reference populations from AFR, AMR, EAS, EUR and SAS. The analysis was conducted by ASA.

Some outliers in the admixed populations were detected by ancestral spectrum analyses using five reference populations [24]. The EAS and SAS ancestries in the admixed populations from the 1KGP are typically small, with average ancestral information of 0.004 and 0.004 in the AA population and 0.007 and 0.005 in the American population, respectively. However, HG01880 from ACB was shown to have a large amount of SAS ancestry, with SAS ancestral information 0.363. HG01944 from PEL has substantial EAS ancestry, with EAS ancestral information 0.377 [24]. Because the EAS ancestry in HG01944 was estimated by the five reference populations that included AMRs, EAS ancestry in AMRs from ancient admixtures was not included. The local ancestry results of individuals HG01880 and HG01944 are displayed in Figure 8 and Figure 9, respectively. As in the previous analyses, AFR, AMR, EAS, EUR and SAS represented the ancestral populations of the two outliers well. The ASA results in the two figures are approximately identical to those obtained by RFMix, G-Nomix and FLARE; the latter can be found in the Supplementary Files. The local ancestry results confirmed the outliers identified by ancestral spectrum analyses that used sparse population-specific SNPs across autosomes.

### 3.4. Computational Time

**Figure 10:**
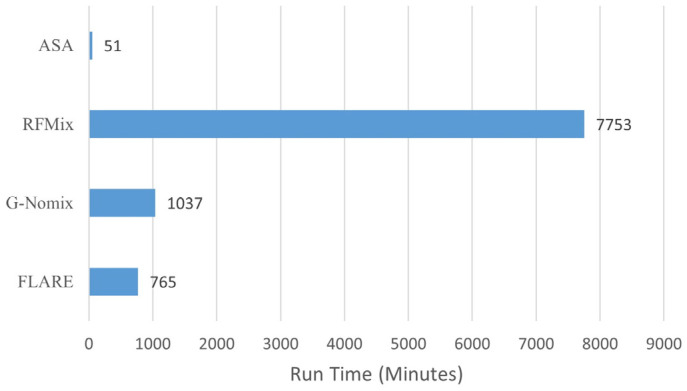
Computational time for ASA, RFMix, G-Nomix and FLARE.

LAI methods are usually computationally extensive, especially for RFMix [4]. We recorded computational runtimes for the four methods in this analysis; their comparison is shown in Figure 10. All computations were performed on a Linux server with two Intel Xeon E5-2650 v3 @ 2.30 GHz, 256 GB memory and the CentOS Linux 8 operating system. The four methods were run one at a time; ASA was run on a single thread, and RFMix, G-Nomix and FLARE were on a maximum of 30 threads. As we can see, ASA is extremely computationally efficient, and RFMix is the most computationally expensive one. ASA on a single thread is at a fraction of the computational time of existing methods on 30 threads, even compared with FLARE. This is because our LAIV involves only counting population-specific alleles and arithmetic operations with allele frequencies.

## 4. Discussion

We conducted local ancestry inferences based on a panel of SNPs that are specific to five reference populations. We showed that the existence of alleles in a chromosomal region that are specific to a particular reference population and the absence of alleles specific to the other reference populations provide reasonable evidence for the coancestry between the region and the reference population. If reference populations approximate ancestral populations of the admixed sample well, the ancestral origin of the region can be determined. The results from RFMix, G-Nomix and FLARE largely agree with those from ASA when the admixtures are recent, in which case contemporary populations serve as proxies for the ancestral populations well. When the admixtures are ancient and contemporary reference populations do not satisfactorily approximate ancestral populations, the performances of RFMix, G-Nomix and FLARE deteriorate with increased error rates and fragmented chromosomal segments. In contrast, ASA provides reasonable results.

ASA is limited by the number of population-specific SNPs available for the reference populations and the number of population-specific alleles carried by the individual under analysis. In the panel of population-specific SNPs, only 55,413 SNPs were specific to AMR populations, which is approximately 1% of the SNPs specific to AFRs. For chromosomal regions of equal physical length, those of AFR ancestry would contain many more AFR alleles than AMR alleles in regions of AMR ancestry. As demonstrated in Figure 3, some regions that originated from AMR cannot be called by ASA properly due to the lack of AMR alleles in these regions. As a result, the average uncalled rate in Americans is 0.066, which is much greater than the 0.012 in AAs because American populations have much greater AMR ancestry than AA populations in the 1KGP [24].

ASA is subject to misclassification errors that may exist in the panel of population-specific SNPs [24]. Alleles that are more frequent in one reference population than the others may be considered specific to the first population if they do not occur in the samples of other reference populations. This error rate can be reduced if the sample sizes of the reference populations increase. We expected that the number of misclassified SNPs in the panel would be small and that their impact on local ancestry inference would also be small. As an example, HG02052 from ACB, who is assumed to have AFR and EUR ancestries only, carries 344,868, 2, 25, 240, and 28 alleles specific to AFR, AMR, EAS, EUR, and SAS, respectively, on 22 autosomes. Suppose that the AMR, EAS and SAS alleles are due to misclassification errors and that the AFR and EUR alleles are of no misclassification errors. A naive estimate of the misclassification error rate is 0.17% after adjusting for different SNP numbers in the panel. Furthermore, alleles due to the misclassification errors typically distribute sporadically on chromosomes and may affect local ancestry inference only if no alleles specific to the true ancestry exist in the window under analysis, which is uncommon. When analyzing with ASA, the option “--allele-min” can be further used to control the impact of misclassification errors, which specify the minimum number of ancestral alleles required to call the ancestry if the window includes alleles specific to more than one population.

We used a sliding window of size 2 M bps in the ASA analysis due to the low density of AMR SNPs. The window size is much larger than the default window sizes used in RFMix and G-Nomix. A large window size allows the inclusion of more population-specific alleles in the analysis and typically produces smooth and statistically reliable results. This will, however, reduce the accuracy on the boundaries of chromosomal segments where ancestry changes. Segments with small physical lengths may not be detected when the window contains alleles from adjacent segments that have a different ancestral origin. Using a smaller window size will yield more accurate results on the boundaries and improve the chance of discovering smaller chromosomal segments. However, the uncalled rate will be larger. Considering that AAs and Americans in the 1KGP have recently been admixed and that their admixture linkage disequilibrium is expected to extend in wide chromosomal regions, the downside of using a large sliding window should be limited.

Most existing methods for local ancestry inference were developed for analyzing genotype data generated by SNP arrays, which are primarily common SNPs, and the typical minimum MAF for inclusion in the analyses is 0.005 [6]. We used a smaller threshold of 0.001 in this work to include rarer SNPs that are population-specific but have MAFs lower than 0.005 in the total population. The analyses by ASA, RFMix, G-Nomix and FLARE were conducted based on the exact same haplotype data after the MAF filtering; hence, comparisons of the four methods were justifiable. We observed that including rarer SNPs is not only necessary for ASA analysis but also beneficial for RFMix, G-Nomix and FLARE analyses. Using the MAF threshold of 0.005, which yielded 11,674,569 autosomal SNPs, we conducted additional analyses with ASA, RFMix, G-Nomix and FLARE. We found that the consistency rates among the four methods were better when rarer SNPs were included. It seems that RFMix, G-Nomix and FLARE somehow exploit valuable ancestral information enriched in population-specific SNPs in their own manners.

## Figures and Tables

**Table 1:** Average consistency rates among ASA, RFMix, G-Nomix and FLARE in AA and American populations. Lower left of the table shows the average consistency rates in AAs, and upper right in Americans.

Method	ASA	RFMix	G-Nomix	FLARE
ASA	1	0.886	0.889	0.898
RFMix	0.955	1	0.925	0.948
G-Nomix	0.964	0.978	1	0.935
FLARE	0.961	0.980	0.984	1

## Data Availability

Genotype data from high coverage sequencing data of the 1KGP are available in the public domain: ftp://ftp.1000genomes.ebi.ac.uk/vol1/ftp/data_collections/1000G_2504_high_coverage, accessed on 15 June 2024. Genotype data from high-coverage sequencing data of the HGDP are available in the public domain: ftp://ngs.sanger.ac.uk/production/hgdp/hgdp_wgs.20190516/, accessed on 15 June 2024.

## Supplementary Materials

The following supporting information can be downloaded at https://www.mdpi.com/article/10.3390/genes15081099/s1: Figure S1: Pipeline to conduct LAI analyses with ASA, RFMix, G-Nomix and FLARE. Figure S2: Consistency rates among ASA, RFMix, G-Nomix and FLARE in ACB population, with reference populations from AFR, AMR, EAS, EUR and SAS. Figure S3: Consistency rates among ASA, RFMix, G-Nomix and FLARE in ASW population, with reference populations from AFR, AMR, EAS, EUR and SAS. Figure S4: Consistency rates among ASA, RFMix, G-Nomix and FLARE in CLM population, with reference populations from AFR, AMR, EAS, EUR and SAS. Figure S5: Consistency rates among ASA, RFMix, G-Nomix and FLARE in MXL population, with reference populations from AFR, AMR, EAS, EUR and SAS. Figure S6: Consistency rates among ASA, RFMix, G-Nomix and FLARE in PEL population, with reference populations from AFR, AMR, EAS, EUR and SAS. Figure S7: Consistency rates among ASA, RFMix, G-Nomix and FLARE in PUR population, with reference populations from AFR, AMR, EAS, EUR and SAS. The Supplementary Files for this article can be found online at https://doi.org/10.6084/m9.figshare.26027158: The panel of population-specific SNPs; Local ancestry results for the 504 individuals with ASA, RFMix, G-Nomix and FLARE.
