# Supplementary material for: Local Ancestry Inference Based on Population-Specific Single-Nucleotide Polymorphisms—A Study of Admixed Populations in the 1000 Genomes Project
Source: Genes (Basel). 2024 Aug 21;15(8):1099. doi: 10.3390/genes15081099 (PMC11353365; doi:10.3390/genes15081099)
Supplement: Supplementary file 1 [file genes-15-01099-s001.zip › genes-3106542-supplementary.pdf]

Local Ancestry Inference Based on Population-Specific Single-Nucleotide Polymorphisms

—— Supplementary Figures

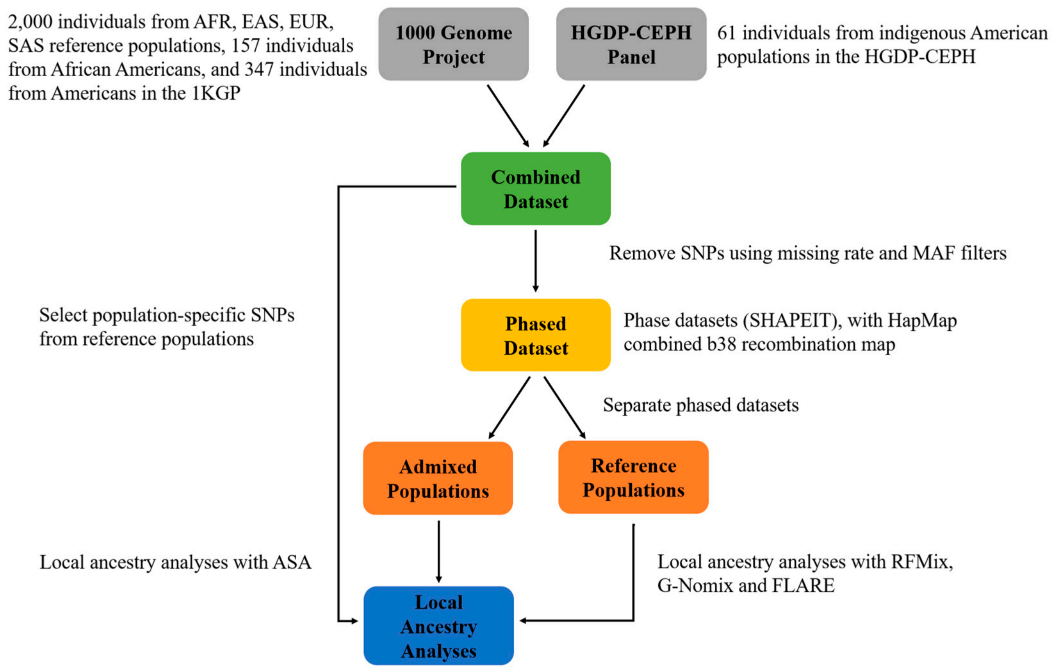

Figure S1. Pipeline to conduct LAI analyses with ASA, RFMix, G-Nomix and FLARE.

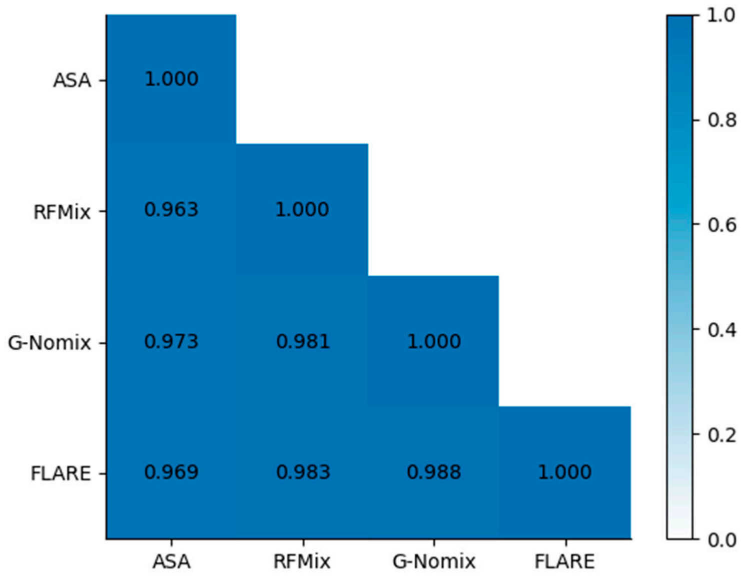

Figure S2. Consistency rates among ASA, RFMix, G-Nomix and FLARE in ACB population, with reference populations from AFR, AMR, EAS, EUR and SAS.

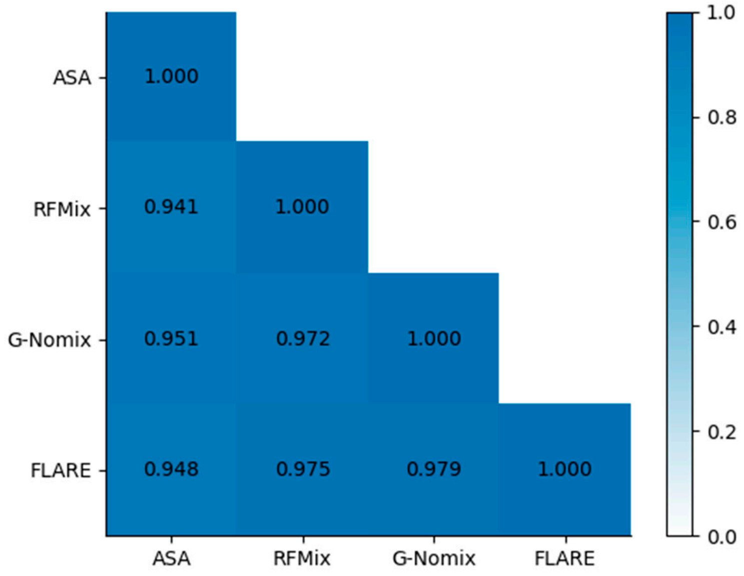

Figure S3. Consistency rates among ASA, RFMix, G-Nomix and FLARE in ASW population, with reference populations from AFR, AMR, EAS, EUR and SAS.

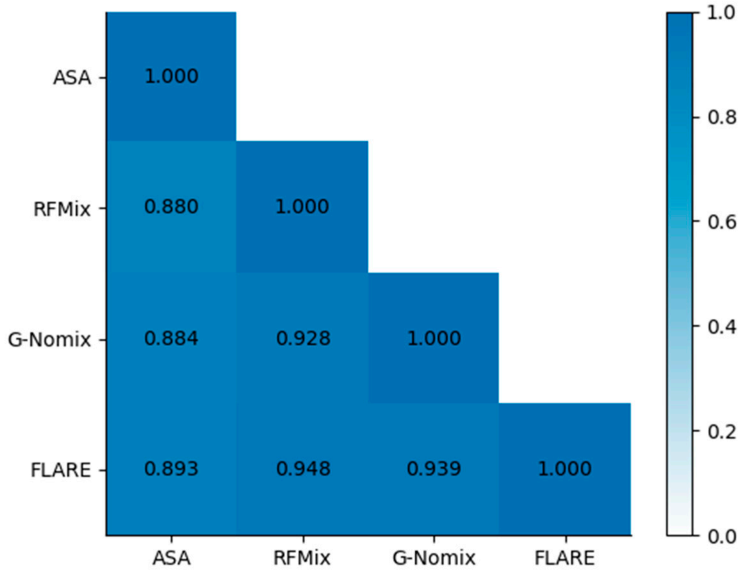

Figure S4. Consistency rates among ASA, RFMix, G-Nomix and FLARE in CLM population, with reference populations from AFR, AMR, EAS, EUR and SAS.

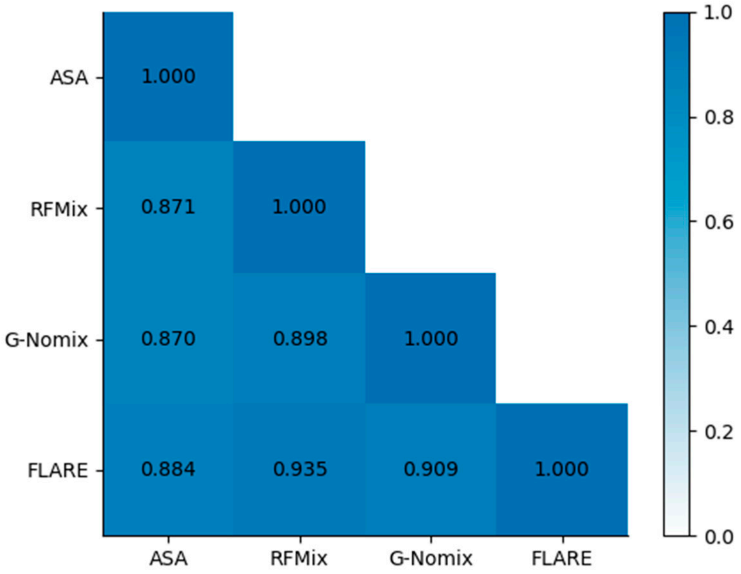

Figure S5. Consistency rates among ASA, RFMix, G-Nomix and FLARE in MXL population, with reference populations from AFR, AMR, EAS, EUR and SAS.

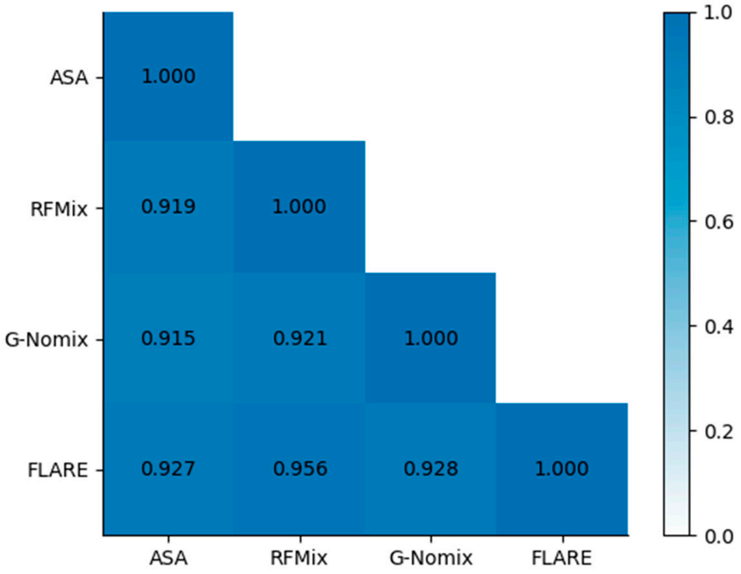

Figure S6. Consistency rates among ASA, RFMix, G-Nomix and FLARE in PEL population, with reference populations from AFR, AMR, EAS, EUR and SAS.

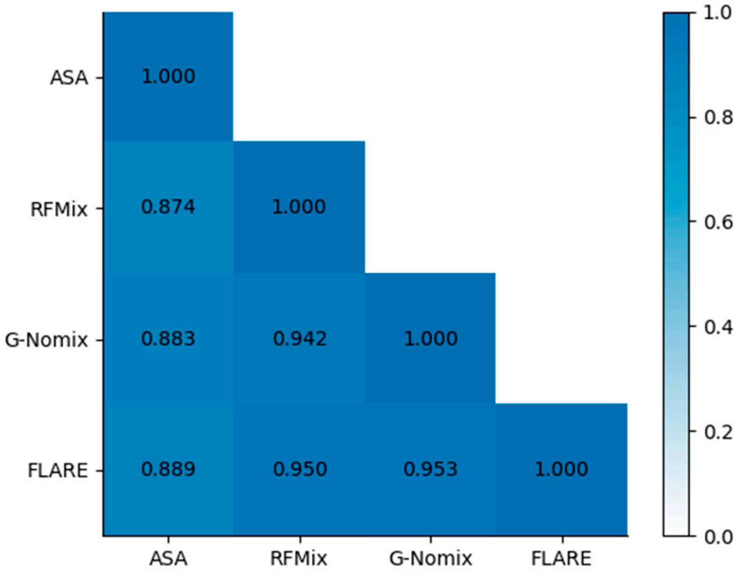

Figure S7. Consistency rates among ASA, RFMix, G-Nomix and FLARE in PUR population, with reference populations from AFR, AMR, EAS, EUR and SAS.
